# “Polymer Analysis” Section, in Journal Polymers

**DOI:** 10.3390/polym12112748

**Published:** 2020-11-20

**Authors:** Giulio Malucelli

**Affiliations:** Department of Applied Science and Technology, and Local INSTM Unit, Viale Teresa Michel 5, 15121 Alessandria, Italy; giulio.malucelli@polito.it

Dear colleagues and friends,

The year 2020 is almost at an end, and our Polymer Analysis section (https://www.mdpi.com/journal/polymers/sections/Polymer_Analysis) has experienced another fruitful year of high-quality Special Issues and scientific papers devoted to different topics of polymer analysis.

Generally speaking, the “Polymer Analysis” section of *Polymers* strives to provide an open-access platform for disseminating high-quality articles and reviews at the core of polymer characterization. In this respect, the “Polymer Analysis” section invites studies on the characterization polymer-based systems (such as polymers, copolymers, and micro- and nano-polymer composites, just to name a few) in order to establish strong structure–property relationships. These characterizations can potentially lead both to a deep knowledge of the materials’ performances and to the possibility of choosing the best performing systems for specific (advanced) applications.

Furthermore, the total number of downloads/views of manuscripts published in *Polymers* in 2020 was 1,190,933/1,489,764, which, for the total of 2670 articles published in 2020 (data till 18th November 2020), implies approximately 558 views per article, demonstrating a superb visibility among both researchers and the scientific community. Finally, in terms of publication time, *Polymers* is among the fastest in the field, with an average of 32 days from submission to first publication. The rapid publishing time, while maintaining a rigorous peer-review process, is undeniably a credit to the dedication and professionalism of the Editors and the Editorial Team of *Polymers*.

It is clear that (1) a favorable and steadily increasing impact factor (IF = 3.426 (2019); 5-year impact factor: 3.636 (2019)), (2) high visibility in open-access publishing, (3) rapid publication times, and (4) the high quality of the rigorous peer-review process are among the benefits of publishing in the “Polymer Analysis” section of *Polymers*.

Besides, there are some additional benefits. (1) We encourage authors and readers to take advantage of Special Issues that focus on a specific area of polymer analysis (at present the “Polymer Analysis” section has run 42 Special Issues) and enjoy record visibility and higher readership than regular issues. (2) Finally, in the era of modern publishing with on-the-go mobile access, the Editorial Team at *Polymers* ensures that the published research enjoys rapid dissemination through a variety of platforms to reach everyone and maximize access. This includes Twitter (@Polymers_MDPI), Facebook, and LinkedIn, with the launch of regular highlights of “Hot Papers” and “Editors’ Choice” manuscripts.

A selection of cutting-edge “Hot Papers” published in the Polymer Analysis section of *Polymers* is presented at the end of this Editorial [1,2,3,4,5,6,7,8,9,10,11,12].

Polymer analysis is experiencing rapid growth. With the improvements in the design and synthesis of new polymer systems, characterization and analytical techniques, development of new technologies, and novel micro- and nano-composite systems, the prospective contribution of polymer analysis research to the overall growth of polymer science and technology seems limitless. We hope that you will consider submitting your polymer analysis papers to *Polymers*.

I hope that in 2021, the Polymer Analysis section will continue to grow and expand with the help of all the Editorial Board, the Editorial Staff, and (last but not least) all the readers and authors!

When submitting polymer analysis papers to *Polymers*, “Polymer Analysis Section” should be selected in the scroll-down submission menu.

“Hot Articles” in the Polymer Analysis section of *Polymers*:

1. Thermal and Calorimetric Evaluations of Some Chemically Modified Carbohydrate-Based Substrates with Phosphorus-Containing Groups (http://www.mdpi.com/2073-4360/12/3/588).
2. Kinetics and Thermodynamics of Thermal Degradation of Different Starches and Estimation the OH Group and H_2_O Content on the Surface by TG/DTG-DTA (http://www.mdpi.com/2073-4360/12/2/357).
3. Poly(lactic Acid)–Biochar Biocomposites: Effect of Processing and Filler Content on Rheological, Thermal, and Mechanical Properties (http://www.mdpi.com/2073-4360/12/4/892).
4. Incentives of Using the Hydrodynamic Invariant and Sedimentation Parameter for the Study of Naturally- and Synthetically-Based Macromolecules in Solution (http://www.mdpi.com/2073-4360/12/2/277).
5. Pyrolysis of Low Density Polyethylene: Kinetic Study Using TGA Data and ANN Prediction (http://www.mdpi.com/2073-4360/12/4/891).
6. Zwitterion Co-Polymer PEI-SBMA Nanofiltration Membrane Modified by Fast Second Interfacial Polymerization (http://www.mdpi.com/2073-4360/12/2/269).
7. Fabrication and Optimization of the Thermo-Sensitive Hydrogel Carboxymethyl Cellulose/Poly(N-isopropylacrylamide-co-acrylic acid) for U(VI) Removal from Aqueous Solution (http://www.mdpi.com/2073-4360/12/1/151).
8. A Comprehensive Review on Water Diffusion in Polymers Focusing on the Polymer–Metal Interface Combination (http://www.mdpi.com/2073-4360/12/1/138).
9. Effect of Bis (2-Aminoethyl) Adipamide/Adipic Acid Segment on Polyamide 6: Crystallization Kinetics Study (http://www.mdpi.com/2073-4360/12/5/1067).
10. Zwitterionic Polymer Brush Grafted on Polyvinylidene Difluoride Membrane Promoting Enhanced Ultrafiltration Performance with Augmented Antifouling Property (http://www.mdpi.com/2073-4360/12/6/1303).
11. Improved Hydrophobicity and Dimensional Stability of Wood Treated with Paraffin/Acrylate Compound Emulsion through Response Surface Methodology Optimization (http://www.mdpi.com/2073-4360/12/1/86).
12. Acrylic Bone Cement Incorporated with Low Chitosan Loadings (http://www.mdpi.com/2073-4360/12/7/1617).

## References

[1] Thomas A., Joseph P., Moinuddin K., Zhu H., Tretsiakova-McNally S., Morgan A.B., Gilman J.W. (2020). Thermal and Calorimetric Evaluations of Some Chemically Modified Carbohydrate-Based Substrates with Phosphorus-Containing Groups. Polymers.

[2] Pigłowska M., Kurc B., Rymaniak L., Lijewski P., Fuć P. (2020). Kinetics and Thermodynamics of Thermal Degradation of Different Starches and Estimation the OH Group and H_2_O Content on the Surface by TG/DTG-DTA. Polymers.

[3] Arrigo R., Bartoli M., Malucelli G. (2020). Poly(lactic Acid)–Biochar Biocomposites: Effect of Processing and Filler Content on Rheological, Thermal, and Mechanical Properties. Polymers.

[4] Grube M., Cinar G., Schubert U.S., Nischang I. (2020). Incentives of Using the Hydrodynamic Invariant and Sedimentation Parameter for the Study of Naturally- and Synthetically-Based Macromolecules in Solution. Polymers.

[5] Dubdub I., Al-Yaari M. (2020). Pyrolysis of Low Density Polyethylene: Kinetic Study Using TGA Data and ANN Prediction. Polymers.

[6] Chiao Y.H., Patra T., Yap Ang M.B.M., Chen S.T., Almodovar J., Qian X., Wickramasinghe S.R., Hung W.S., Huang S.H., Chang Y. (2020). Zwitterion Co-Polymer PEI-SBMA Nanofiltration Membrane Modified by Fast Second Interfacial Polymerization. Polymers.

[7] Tan J., Xie S., Wang G., Yu C.W., Zeng T., Cai P., Huang H. (2020). Fabrication and Optimization of the Thermo-Sensitive Hydrogel Carboxymethyl Cellulose/Poly(N-isopropylacrylamide-co-acrylic acid) for U(VI) Removal from Aqueous Solution. Polymers.

[8] Yang C., Xing X., Li Z., Zhang S. (2020). A Comprehensive Review on Water Diffusion in Polymers Focusing on the Polymer–Metal Interface Combination. Polymers.

[9] Chen Y.H., Ranganathan P., Chen C.W., Lee Y.H., Rwei S.P. (2020). Effect of Bis (2-Aminoethyl) Adipamide/Adipic Acid Segment on Polyamide 6: Crystallization Kinetics Study. Polymers.

[10] Chiao Y.H., Chen S.T., Sivakumar M., Yap Ang M.B.C., Patra T., Almodovar J., Wickramasinghe S.R., Hung W.S., Lai J.Y. (2020). Zwitterionic Polymer Brush Grafted on Polyvinylidene Difluoride Membrane Promoting Enhanced Ultrafiltration Performance with Augmented Antifouling Property. Polymers.

[11] Jiang J., Chen Y., Cao J., Mei C. (2020). Improved Hydrophobicity and Dimensional Stability of Wood Treated with Paraffin/Acrylate Compound Emulsion through Response Surface Methodology Optimization. Polymers.

[12] Valencia Zapata M.E., Mina Hernandez J.H., Grande Tovar C.D. (2020). Acrylic Bone Cement Incorporated with Low Chitosan Loadings. Polymers.

